# From across the Seas

**Published:** 1920-02-14

**Authors:** 


### FROM ACROSS THE SEAS.

### A South Australian Hospital.

THE annual report of Adelaide Hospital, South Australia, has reached us, and contains, amongst other interesting features, excellent photographs of the buildings and the sub-tropical grounds in which they are situated. The Nurses' Home is a particularly handsome building, and possesses commodious balconies on all floors with verandas on the first and second floors. The board of management, it is stated, has been much exercised lately by grave signs of proselytising, and has had to make strict rules under which no clergyman or representative of any church is allowed to hold any conversation with any patient unless that patient is a member or adherent of the church represented by the spiritual adviser. This embargo, however, does not apply to intercourse between ministers and patients when they are personal friends.

### The American Hospital Association.

THE next annual conference of the American Hospital Association will take place within the British Dominions, as Montreal has been chosen as the place of meeting. Apart from the actual business of the conference there will be plenty to interest the members, with the Royal Victoria and Montreal General Hospitals within the city and the magnificent new buildings of the McGill University within easy reach. The new executive secretary of the association is Dr. Andrew Robert Warner, who for a number of years was superintendent of Lakeside Hospital, Cleveland, Ohio. Dr. Warner began his duties on November 1, and succeeds Mr. Howell Wright, who for about a year was acting as temporary executive secretary. Dr. Warner's appointment is very popular, and his activity in hospital matters generally has won him many friends in the hospital world.

